# Editorial: Insights into gastrointestinal cancer metastasis from preclinical models

**DOI:** 10.3389/fonc.2026.1865161

**Published:** 2026-05-20

**Authors:** Bruna Costa, Alyssa Schledwitz, Jean-Pierre Raufman

**Affiliations:** 1 Champalimaud Centre for the Unknown, Champalimaud Foundation, Lisbon, Portugal; 2 University of Maryland School of Medicine, Division of Gastroenterology and Hepatology, Baltimore, MD, United States

**Keywords:** gastrointestinal cancer (GI cancer), metastasis, non-coding RNAs, preclinical models, translational oncology

For some 40 years or so, mice have served as the primary preclinical model for scientific studies, including work focused on elucidating the mechanisms underlying the spread of cancer cells from primary tumors to other organs. Metastatic spread of neoplastic cells beyond the primary site is the major driver of gastrointestinal (GI) cancer death; patients rarely die from the primary lesion. Several factors favored the mouse model for preclinical studies of neoplasia, including the ability to manipulate the murine genome to create whole body or conditional knockouts, to over-express selected genes, or to perform surgical, chemical, or other manipulations that cannot be conducted in humans. With specific relevance to colon cancer, the ability to mimic human colon carcinogenesis in murine models by genetic manipulations (e.g., *Apc^Min/+^* mice) or chemical induction (e.g., azoxymethane-treatment) was a prominent advantage despite the disadvantage that additional genetic or surgical manipulations were required to induce metastatic spread of cancer beyond the colon. Of course, the need for such manipulations in mice that are not needed for humans to develop colon neoplasia raised reasonable questions regarding whether these murine models faithfully replicate gastrointestinal neoplasia in humans.

Notably, although much useful mechanistic information was gleaned from murine studies, investigators and others have raised important concerns regarding the relevance of experimental findings derived from such studies to human physiology and disease. Several analyses have revealed the unfortunate truth that some 80% of novel therapeutic approaches that showed promise in mouse studies, failed to demonstrate benefit when tested in human clinical trials. At the same time, for ethical and other concerns, there has been a growing movement towards the use of non-mammalian experimental models, including the application of organoids, zebrafish xenografts, as well as the promise of preclinical models employing artificial intelligence. Hence, we believe this Frontiers in Oncology Research Topic, Insights into Gastrointestinal Cancer Metastasis from Preclinical Models, is timely and relevant to key questions facing those involved in cancer research.

Progress in understanding gastrointestinal cancer metastasis has been linked with the development of experimental models that better capture its biological complexity. There has been increasing interest in understanding and dissecting the complex interactions within the tumor microenvironment of cancer cells with immune cells, neurons, enteroendocrine cells, and gastrointestinal luminal molecules (e.g., bile acids) and a variety of microorganisms and their metabolic byproducts (metabolome) within the gut microbiome. Likewise, there has been increasing recognition of the great diversity of the cells that comprise gastrointestinal tumors – findings facilitated by advances in single cell RNA sequencing and other technological advances. These newer model systems have made it possible to move beyond descriptive observations and start dissecting the mechanisms that drive metastatic behavior, from tumor-intrinsic regulatory circuits to interactions with other cells within the tumor microenvironment. The studies in this Research Topic reflect this transition, highlighting how model systems can uncover clinically relevant drivers of metastasis.

A common theme emerging from these contributions is the role of non-coding RNAs as central regulators of metastatic progression. Acting within interconnected regulatory networks, these molecules link intracellular signaling with intercellular communication, shaping both tumor cell behavior and microenvironmental dynamics.

Xiao et al. identify a circular RNA, circRERE(4–5), as a driver of gastric cancer progression. Their data show that circRERE(4–5) is elevated in tumor tissues and patient plasma, with levels correlating with tumor burden and metastatic spread. Mechanistically, circRERE(4–5) functions as a competing endogenous RNA, sequestering miR-571 and promoting expression of the transcription factor ONECUT2, a known regulator of tumor growth and microenvironmental interactions. Using *in vivo* xenograft models, the authors demonstrate that antisense oligonucleotide-mediated silencing of circRERE(4–5) significantly reduces tumor growth and metastasis without evident toxicity. These findings support the therapeutic potential of targeting circRNA-driven pathways and illustrate how preclinical models can bridge mechanistic insight and translational application.

In contrast, Cao et al. focus on the tumor microenvironment, examining how miR-19b-3p mediates communication between colorectal cancer cells and tumor-associated endothelial cells. Their results show that miR-19b-3p promotes epithelial-to-mesenchymal transition in tumor cells while inducing endothelial-to-mesenchymal transition in endothelial cells, pointing to a coordinated mechanism that enhances metastatic potential.

Clinically, elevated circulating miR-19b-3p is associated with advanced disease and poor prognosis, supporting its potential as both a biomarker and a therapeutic target. These findings emphasize the importance of targeting not only tumor cells but also the microenvironmental interactions that support disease progression.

Both studies show that non-coding RNAs integrate metastatic signaling across cellular compartments. More broadly, these contributions reflect a shift toward functionally driven and translational research. By combining patient-derived data with mechanistic studies and *in vivo* validation, they illustrate how experimental models can be used to identify clinically relevant vulnerabilities.

Many challenges remain, including understanding how these regulatory networks vary across GI cancer types and how they interact with other layers of tumor biology. Emerging technologies, including single-cell omics, high-resolution imaging, and genome editing, will undoubtedly play a pivotal role in addressing these challenges. In summary, this Research Topic highlights how innovative preclinical models can provide integrated insights into GI cancer metastasis, linking mechanism to potential therapeutic strategies.

